# Culture at a Higher Temperature Mildly Inhibits Cancer Cell Growth but Enhances Chemotherapeutic Effects by Inhibiting Cell-Cell Collaboration

**DOI:** 10.1371/journal.pone.0137042

**Published:** 2015-10-23

**Authors:** Shengming Zhu, Jiangang Wang, Bingkun Xie, Zhiguo Luo, Xiukun Lin, D. Joshua Liao

**Affiliations:** 1 Hormel Institute, University of Minnesota, Austin, Minnesota, 55912, United States of America; 2 Department of Oncology, Taihe Hospital, Hubei University of Medicine, Shiyan, Hubei Province, 442000, P.R. China; 3 Department of Pharmacology, Capital Medical University, 10 West, Youanmen Outside, Beijing, 100069, China; Wayne State University, UNITED STATES

## Abstract

Acute febrile infections have historically been used to treat cancer. To explore the underlying mechanism, we studied chronic effects of fever on cancer cell growth and chemotherapeutic efficacy in cell culture. We found that culturing cancer cells at 39°C mildly inhibited cell growth by arresting the cells at the G1 phase of the cell cycle. When cells were seeded in culture dishes at a lower density, e.g. about 1000–2000 cells per 35-mm dish, the growth inhibition was much greater, manifested as many fewer cell colonies in the 39°C dishes, compared with the results at a higher density seeding, e.g. 20,000 cells per dish, suggesting that cell-cell collaboration as the Allee effect in cell culture is inhibited at 39°C. Withdrawal of cells from serum enhanced the G1 arrest at 39°C and, for some cell lines such as A549 lung cancer cells, serum replenishment failed to quickly drive the cells from the G1 into the S and G2-M phases. Therapeutic effects of several chemotherapeutic agents, including clove bud extracts, on several cancer cell lines were more potent at 39°C than at 37°C, especially when the cells were seeded at a low density. For some cell lines and some agents, this enhancement is long-lasting, i.e. continuing after the cessation of the treatment. Collectively these results suggest that hyperthermia may inhibit cancer cell growth by G1 arrest and by inhibition of cell-cell collaboration, and may enhance the efficacy of several chemotherapeutic agents, an effect which may persist beyond the termination of chemotherapy.

## Introduction

Acute febrile infections by different pathogens have for centuries been thought to play a role in cancer prophylaxis [1,2] and in cancer spontaneous regression [3–7], as reviewed before by us [8] and others [9]. Actually, different pathogens that can cause acute fever, such as bacteria and malaria-causing parasitic protozoa, were already used to treat cancers over a century ago [5,10]. During 1866–1867, Busch in Germany infected sarcoma patients with erysipelas-causing bacteria, which resulted in not only high fever but also the tumor remission within two weeks, and iterations of the procedure prevented regrowth of the tumor [4,11,12]. In 1882, Fehleisen confirmed Busch’s therapy and identified *Streptococcus pyogenes* as the erysipelas-causing bacteria [13]. In 1887, Bruns also cured a recurrent melanoma with erysipelas and summarized 14 reported cases with complete or stable remission [14]. During 1891–1936, Coley at New York injected a bacterial mixture of *S pyogenes* and *Serratia marcescens* [15] into patients with sarcomas or certain epithelial cancers [10]. About 500 of the 1000 patients so treated by Coley and others showed tumor regression [15–18]. Likely, this bacterial mixture, dubbed as “Coley’s vaccine” or “Coley’s toxin”, not only is an immunotherapy [15] but also works through hyperthermia (HT), because its efficacy largely depended on whether the patients responded with higher fevers [10,16]. Actually, HT therapy of cancers acts largely by stimulating immune function, including activation of dendritic cells, natural killer cells and T-cell immune response [19–21]. Moreover, many cancer patients manifest hypothermia or feel “cold” during chemotherapy, possibly because the body mistakes the chemo drug for a toxin and thus lowers the temperature to minimize its “toxicity” [22]. If this conjecture is correct, raising the body temperature may restore the chemo efficacy.

Two important papers published in the mid-1980s have established that a temperature of 42°C for one hour can kill cancer cells while sparing normal cells [23,24] and thus have set a thermal goal to 42–43°C for HT therapy of cancer in most recent studies [25,26]. Many devices have since then been developed and used clinically to treat cancers, aiming to raise the core body temperature to 43–45°C for a duration from 15 minutes to 6 hours [27]. This design of “a short period of high temperature” is also devised because it is not practical to keep the patients in the device for a long time and for many repeated exposures. However, clinical practice has proved that these devices have difficulties in raising the tumor temperature to 42°C. Since there are basically no patients showing a feverish temperature higher than 42°C, 39–42°C becomes the goal in some studies [26]. Stevens et al reported that culture of COLO-357 human pancreatic cancer cells at 42°C increases chromosome fragmentation, a newly identified mitotic cell death, and the induction occurs within 24 hours [28].

Besides a direct thermal kill of cancer cells, HT has also been shown to enhance radio- and chemo-therapies of many cancers, especially the therapies with cisplatin [29–31]. The mechanisms for these efficacy enhancements differ among different chemotherapeutic agents. For cisplatin, HT increases the cell membrane permeability and fluidity that result in cellular accumulation of cisplatin, and increases platinum-DNA adduct formation, while inhibits the repair of cisplatin-caused DNA damage [29–31].

Determining a mild effect of a factor on cell growth in vitro is technically difficult. MTT (3-(4,5-dimethylthiazol-2-yl)-2,5-diphenyltetrazolium bromide) assay or similar colorimetric methods that detect the viable cells by determining the reduction of MTT or a related tetrazolium salt can only detect cell viability for a period of several days. This is because in a 96-well plate the untreated cells included as controls will grow to confluence in a few days, manifested as a plateau of the optical density of reduced MTT. Pertaining to the studies of HT, since cells at a HT temperature and their 37°C controls are seeded in two different 96-well plates for culture in two different incubators, variation is greater compared with the routine MTT assay in which the tested group and its control are in the same plate. Colony growth assay can detect a much longer period of cell growth and provide information on the incremental growth of each initially seeded cell. However, the cells need to be seeded at a much smaller number in the culture dish to avoid fusion of the colonies. For most cell lines, the cells need to be seeded at a certain number or density because cells need to collaborate with each other in order to survive or grow, which is considered as the cell culture version of the Allee effect [32,33]. Therefore to some extent colony growth assay selects those subpopulations of cell survival and growth of which are less dependent on cell-cell collaborations, although this Allee effect *per se* may also be a parameter to evaluate a treatment.

## Materials and Methods

### Human cell lines and reagents

Hela cervical cancer cell line, A549 and H1650 non-small cell lung cancer cell lines, HCT116 colorectal cancer cell line, as well as HEK293T (T large antigen expressing human embryonic kidney) cell line were originally purchased from American Type Cell Culture (ATCC) and used in the study. Dry clove buds were purchased from a Chinese grocery store. To make its ethanol extract, 1 gram of clove buds was added into 5 ml of 95% ethanol in a 15-ml falcon tube and extraction was allowed for two weeks at room temperature [34]. The ethanol was then transferred to a new tube and regarded as a 100% extract for use. In addition, a pure essential oil of clove buds was purchased from NOW Foods Bloomingdale, IL 60108, USA, and was diluted with absolute ethanol. Ethanol was used as the untreated control. Cisplatin and 5-fluorouracil (5-FU) were purchased from Sigma-Aldrich (http://www.sigmaaldrich.com) and diluted with saline or DMSO, respectively.

### Cell culture

Cells were cultured in two water-jacket incubators with the temperature set at 37°C and 39°C, respectively. To ensure the accuracy and stability of the temperature, a thermometer was put in the incubators to monitor the actual temperature. The incubators were supplied with 5% CO_2_ that was recalibrated now and then to ensure the accuracy. During medium change, the culture dishes were taken out by piecemeal from the incubator to minimize the time at the room temperature. The fresh medium was pre-warmed in the corresponding incubator.

### MTT assay

Cells in the logarithmic phase were harvested and seeded into a 96-well plate at a density of 8×10^3^ per well, followed by overnight culture to allow attachment. To determine the effect of 37°C and 39°C cultures, the cells were allowed to grow for additional 72 hours. MTT was then added into the well and, 3 hours later, 200 μL DMSO was added to dissolve the formazan at room temperature for 30 minutes. The optical density of the reduced MTT was measured using a Multiskan-spectrum instrument with a fixed program for MTT assay as reported before [35,36]. To determine the effects of chemotherapeutic drugs, after overnight attachment the cells were treated with a medium containing cisplatin, 5-FU or clove extract at the indicated concentration for 72 hours in the 37°C or 39°C incubator, with relevant solvent as the untreated controls.

### Crystal violet staining

Cells were seeded at an indicated number per dish and cultured in 37°C and 39°C incubators with a RPM-1640 medium containing 5% fetal bovine serum and streptomycin. Medium was changed every 3–4 days or when its color turned slightly yellow. For drug treatment, a medium containing an indicated concentration of cisplatin, 5-FU or clove extract was added. Before staining with crystal violet, the medium was removed and the dish was gently washed with PBS. A fixative (methanol-acetic acid at 3:1 ratio) was added to fix the cells for 30 minutes. The fixative was discarded and the dish was allowed to air-dry completely, followed by staining the cells with a 0.1% crystal violet water solution for 15 minutes. After residual crystal violet was washed away with deionized water, the dish was air-dried and photographed with a digital camera.

### Cell cycle analysis

Cells were cultured in 60-mm dishes until they reached 70~80% confluence. The cells were harvested with gentle trypsin digestion, washed with cold PBS, and then fixed with 70% ethanol in PBS for 30 minutes. The cells were sorted using a Becton Dickinson FACS Calibur flow cytometer (BD Biosciences, San Jose, CA). After the cells were gated in the FSC/SSC plot to exclude small debris, the populations of cells at different stages of the cell cycle were quantified using a ModFit LT software (Verity Software House, Inc., Topsham, ME), as described before [35,37,38]. In each experiment, each group was set mostly in triplet and the data were expressed as the mean±SD (standard deviation).

### Statistical analysis

Most data varied greatly among different repeats of the experiments and were thus analyzed using Wilcoxon rank-sum test. The methods used were indicated in the corresponding figures.

## Results

### HT may cause artifacts in MTT assays

**Fig 1:**
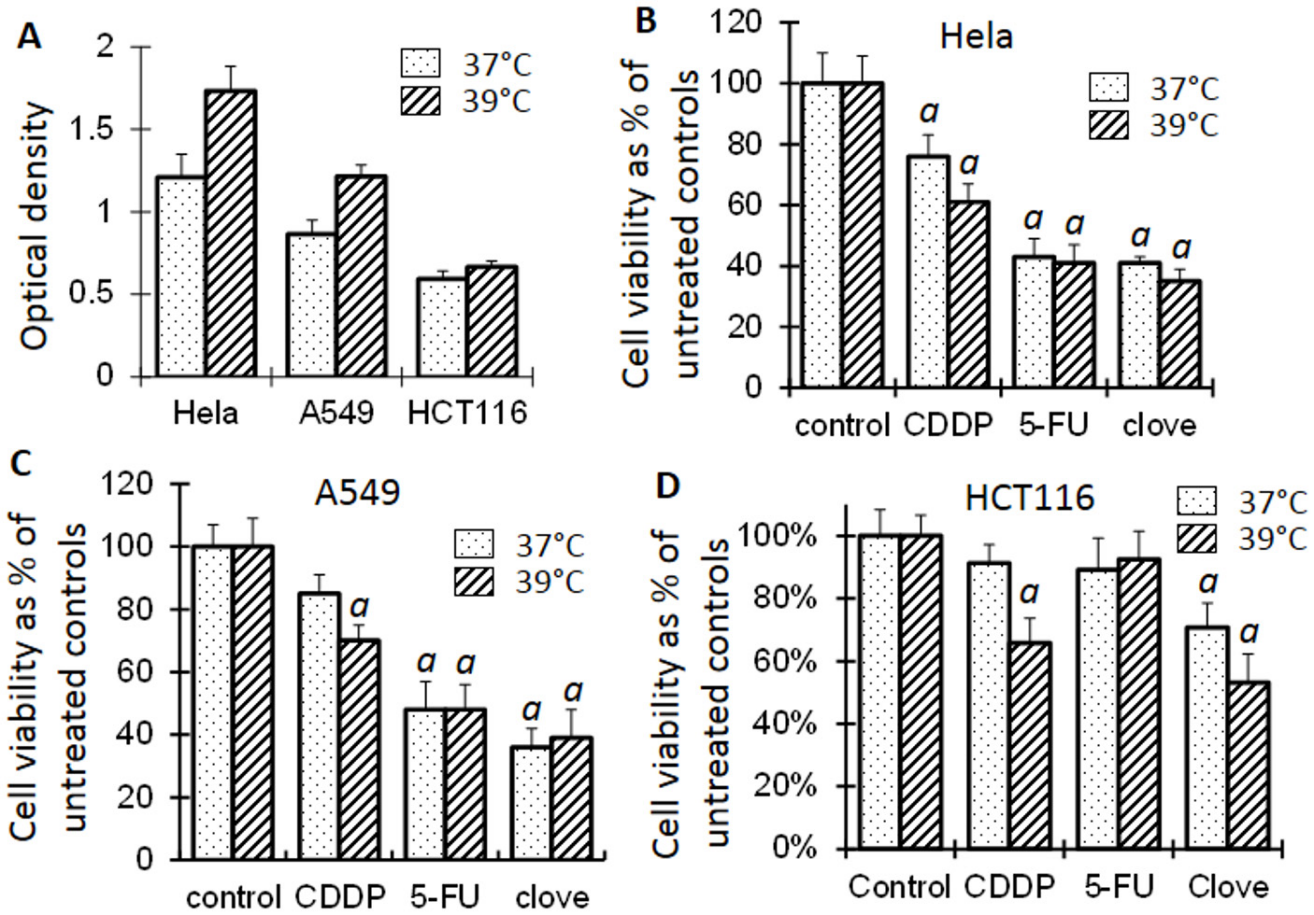
Cell viability detected by MTT assays. **A**: The optical density of MTT is often higher in the cells at 39°C than at 37°C, even when fewer cells at 39°C are observed under microscope, indicating that cell viability may be overestimated at 39°C when MTT assay is used. **B**, **C** and **D**: Treatment of Hela, A549 and HCT116 cells with 1 μM cisplatin (CDDP), 4 μM 5-FU or 0.05% clove bud ethanol extract significantly decreases the cell viability compared with the untreated control at the same temperature (*a* in C, B and D, p<0.05; *t* test), whereas comparison between 37°C and 39°C is not recommended.

We usually observed fewer cells or lower cell densities under microscope, and detected fewer cell colonies using crystal violet staining, in the 39°C dishes than in their 37°C counterparts, but MTT assay often gave rise to an opposite result (Fig 1A). MTT assay determines reduction of MTT, which occurs in the cytosol, mitochondria and even nucleus [39]. Since a higher temperature usually enhances chemical reactions, we tend to conclude that the “higher cell viability” at 39°C detected by an MTT assay is due to an increased activities of some MTT-reducing enzymes and thus is an artifact. Supporting this inference, the culture medium at 39°C turned yellow quicker than at 37°C and needed to be changed more often, indicating a higher metabolic rate at 39°C. Since other colorimetric methods for detection of cell viability actually detect metabolic activities as well [40–42], in most cases with a tetrazolium salt as the substrate [39], currently we still lack a reliable and feasible approach to quantify the effect of HT in culture.

### Cells at 39°C grow slightly slower

**Fig 2:**
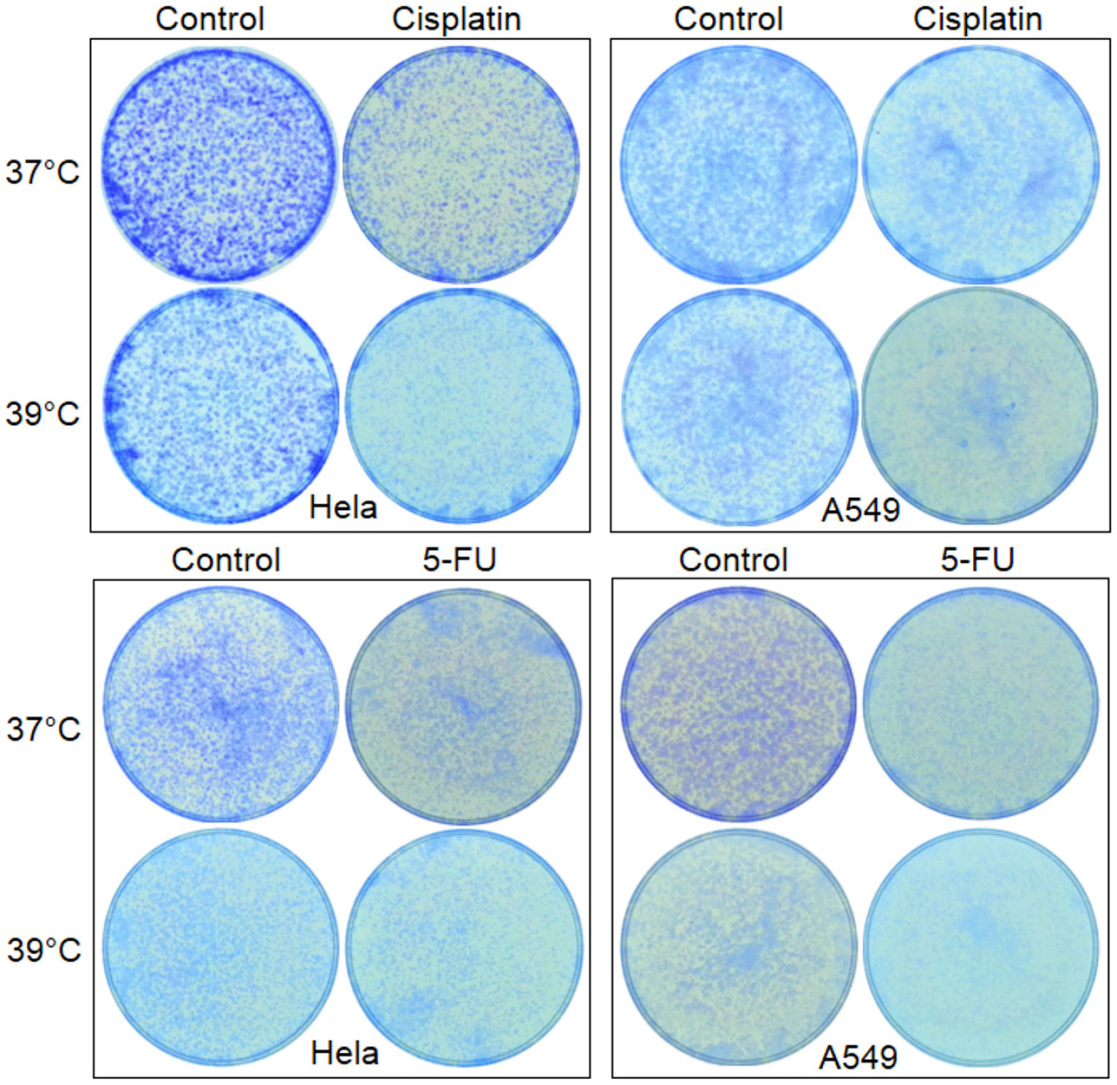
About 20,000 Hela or A549 cells were seeded in each of 35-mm dishes and allowed to grow to about 70% confluence. The cells were then treated with 1 μM cisplatin or 2 μM 5-FU for 3 days and, after the drug was withdrawn, were continued on the culture for 3 additional days so that the cell colonies grow to visible sizes. Note that the cells of solvent-treated control also show a lower density at 39°C than at 37°C.

**Fig 3:**
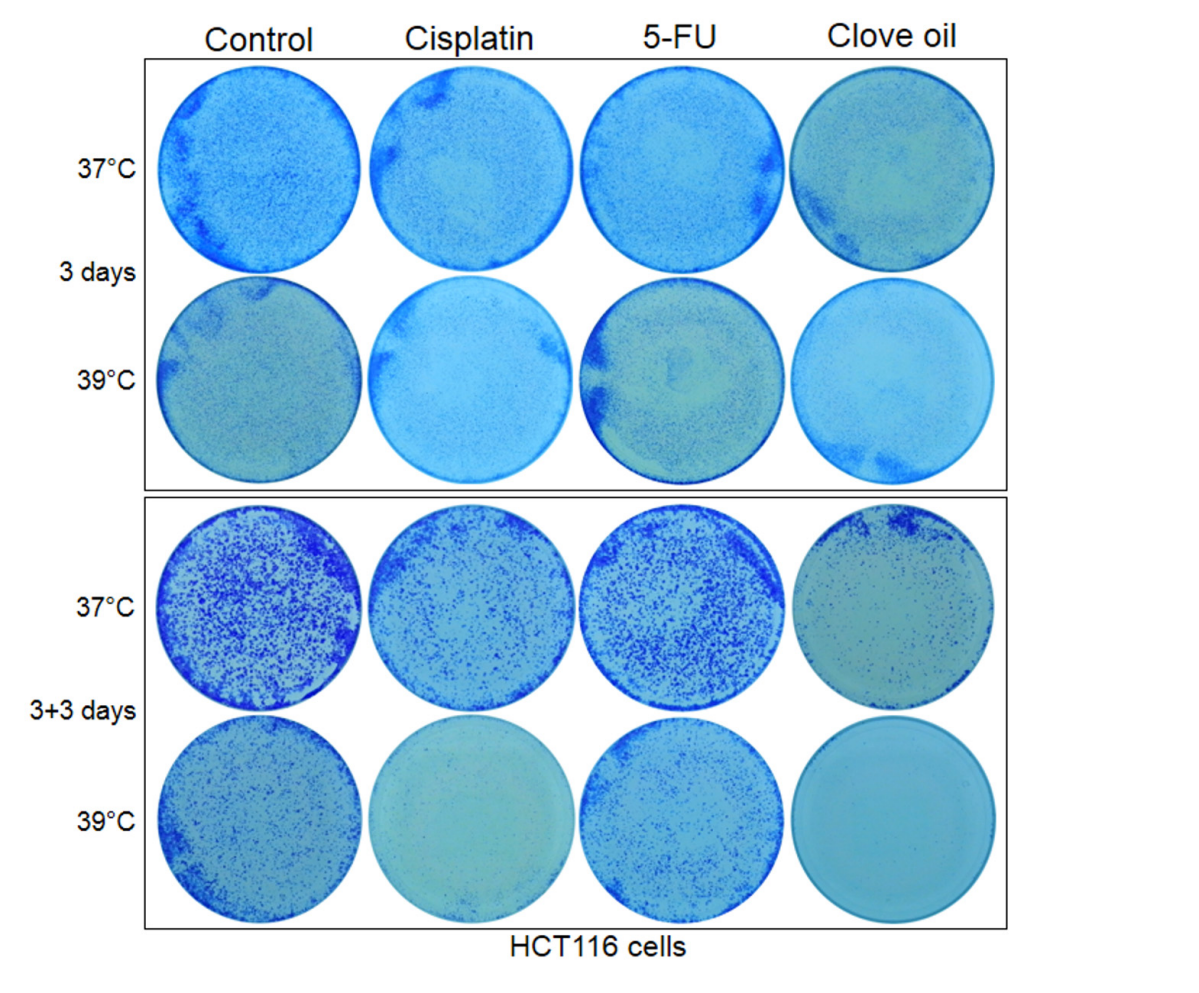
Enhanced effects of cisplatin, 5-FU or clove oil on HCT16 cells at 39°C. **Top panel**: About 20,000 HCT116 cells were seeded in a 35-mm dish and allowed to grow for 3 days at 37°C or 39°C. The cells were then treated with indicated agent for 3 days, followed by staining the viable cells with crystal violet. **Bottom panel**: The cells were treated as above but were allowed to grow for 3 more days after the 3-day treatment with the indicated agent was terminated. Note that the difference in cell density between 37°C and 39°C was widened for cisplatin- and clove-treated cells, compared with the counterpart at the top panel. However, this post-treatment effect is not discerned in 5-FU treated cells at 39°C.

Using an MTT assay in which cells at 37°C and 39°C were seeded in separate 96-well plates, we did not detect obvious difference in the viability of Hela, A549, HCT116, H1650 and HEK293T cells between the two temperatures (not shown), in part due to the technical constraint of MTT assay described above. However, cell density visualized by crystal violet staining was actually lower in the 39°C dishes than in the 37°C ones (Figs 2 and 3; control panels).

**Fig 4:**
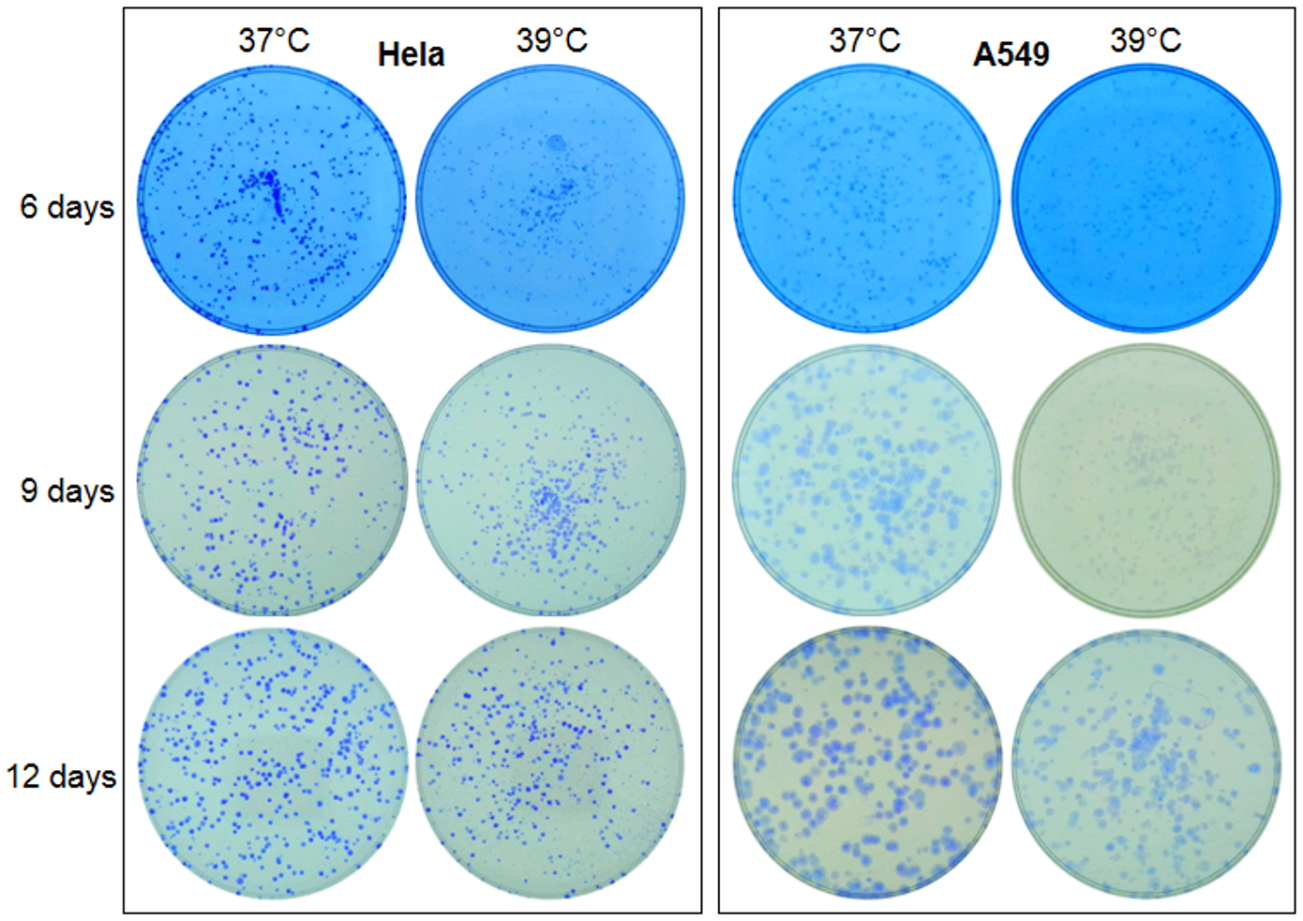
About 1000 Hela or A549 cells were seeded in each of 35-mm dishes and allowed to grow for 6, 9 or 12 days with medium changes. Note that there are fewer colonies of visible sizes in the 39°C dish than in the 37°C one. A549 cells form larger colonies than Hela cells at the later time points, especially at 37°C.

**Fig 5:**
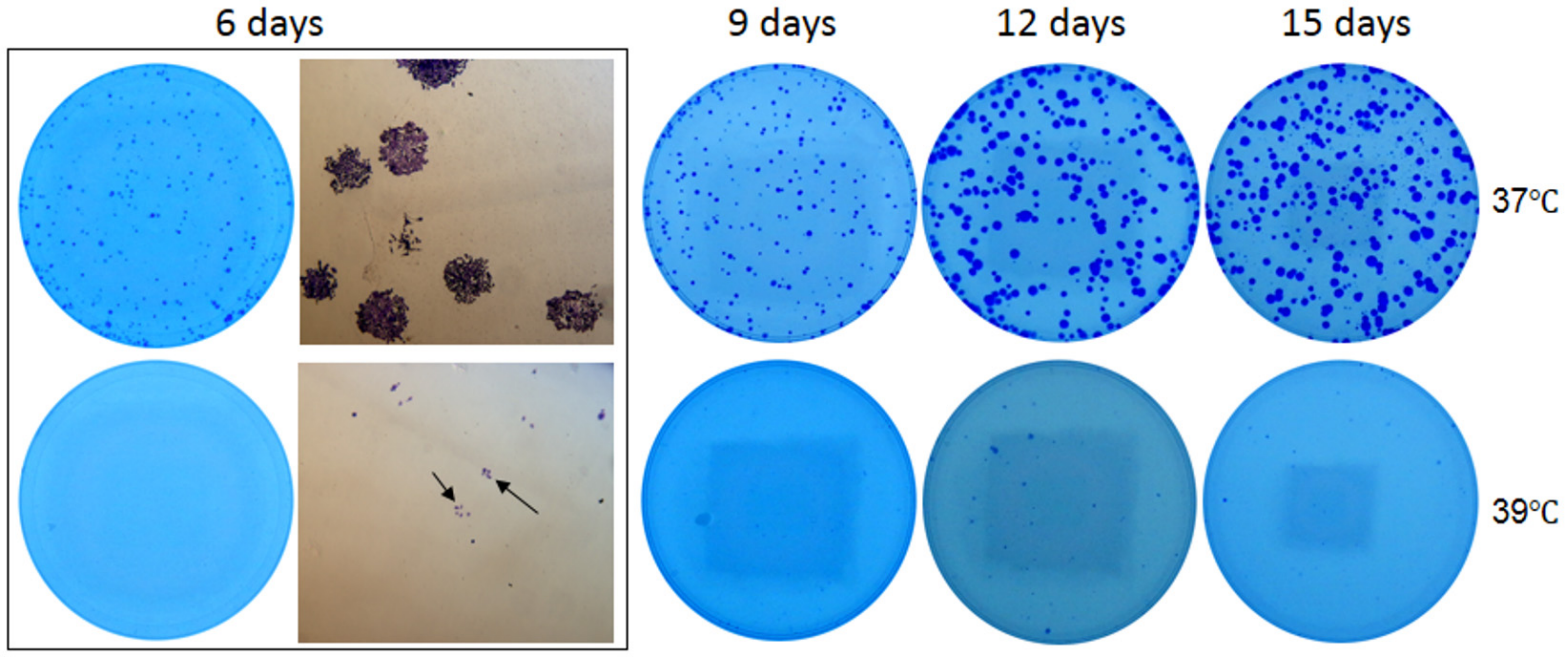
About 1000 HCT116 cells were seeded in each of 35-mm dishes and allowed to grow for the indicated days. Growth is much slower at 39°C than at 37°C, but the cells are still alive, as observed under the microscope (arrows in the 6-day dish).

In order to determine a long-term effect of 39°C on cell growth, we seeded only 1000–2000 cells in a 35-mm dish so that cell colonies would not fuse together. Surprisingly, many fewer colonies of visible sizes were discerned in the 39°C dishes than in the 37°C ones after 6, 9, 12 and 15 days of culture (Figs 4 and 5). However, under microscope there still were colonies that were smaller than the visible size in the 39°C dishes, although the number of these tiny colonies was still smaller compared with the 37°C dishes. These differences, which were more evident for HCT116 cells (Fig 3), indicate that in the 39°C dishes many cells had died while those still-alive had been growth-arrested. We conclude that cells need to collaborate with each other for survival and replication, which is the so-called Allee effect in the dish, and this collaboration is inhibited at 39°C. However, the decrease in the number of viable cells at 39°C, due to either enhanced cell death and/or inhibited cell replication, is so minimal that it could not be discerned in the first three days of culture.

**Fig 6:**
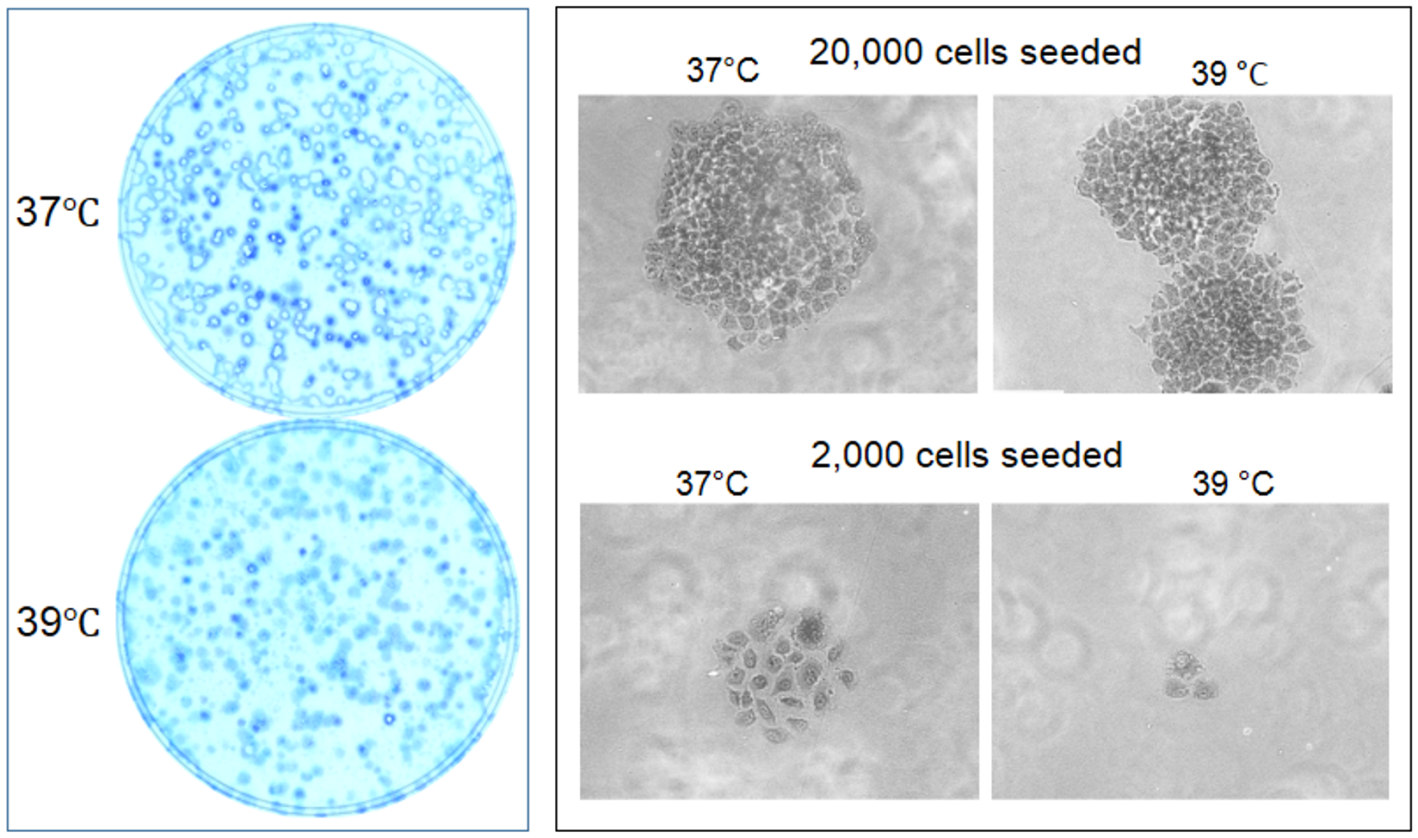
Inhibited growth of Hela cells at 39°C. **Left panel**: Hela cells usually grow in three dimensions in the dish to form spherical colonies which, after growing beyond 9 days, easily shed off during crystal violet staining, leaving the dish with only the periphery of the colonies, especially in the 37°C dish wherein the colonies are much larger. **Right panel**: Six days after the cells were seeded, individual cells seeded at a density of about 20,000 cells per 35-mm dish develop to larger colonies, compared with the colonies in the dish wherein cells were seeded at a density of 2,000 cells. Cells at 37°C develop larger colonies than at 39°C.

Under the microscope, Hela cells replicated in three-dimensions, i.e. could also pile up together, which made the enlargement of the colonies less pronounced in two dimensions. The spherical colonies easily shed off during crystal violet staining, leaving only a periphery of the colonies in the dish (Fig 6). In contrast, A549 cells formed much larger colonies because these cells are larger in cellular size and grew only in two dimensions without piling up together, although they grew much slower than Hela cells (Fig 4).

### Effects of chemotherapeutic agents are potentiated and long-lasting at 39°C

Compared with the corresponding untreated control, treatment with a low concentration of cisplatin (1 μm) for three days slightly decreased the viability of Hela cells, but not that of A549 and HCT116 cells, at 37°C; however, decreases occurred to all three cell lines at 39°C, as detected by MTT assays (Fig 1B, 1C and 1D). Staining of the cells in dishes with crystal violet showed that all three cell lines treated with cisplatin displayed a decreased cell density at 37°C and, more pronouncedly, at 39°C (Figs 2 and 3). Microscopic observation also had the same finding (not shown). Collectively, these results confirm that 39°C potentiates the effect of cisplatin.

MTT assays detected decreased viability of Hela and A549 cells treated with a low concentration (4 μM) of 5-FU (Fig 1B, 1C and 1D). Others and we had previously reported that ethanol extract of clove buds had a potent therapeutic effect on a variety of cancer cell lines [34,43,44]. When treated with the ethanol extract of clove buds, Hela, A549 and HCT116 cells all showed decreased viability as detected with MTT assays (Fig 1B, 1C and 1D). However, crystal violet staining displayed decreased density of Hela, A549 and HCT116 cells treated with 5-FU or clove ethanol extract at 37°C and further showed a more pronounced effect on A549 cells, but showed hardly any effect on Hela and HCT116 cells, at 39°C (Figs 2 and 3). Because ethanol mainly extracts hydrophobic components, we also treated these cell lines with an essential oil of clove buds, which resulted in potent killing effect as well (Fig 3). These results indicate that 39°C indeed potentiates chemo sensitivity but the potentiation is cell-line and agent specific.

**Fig 7:**
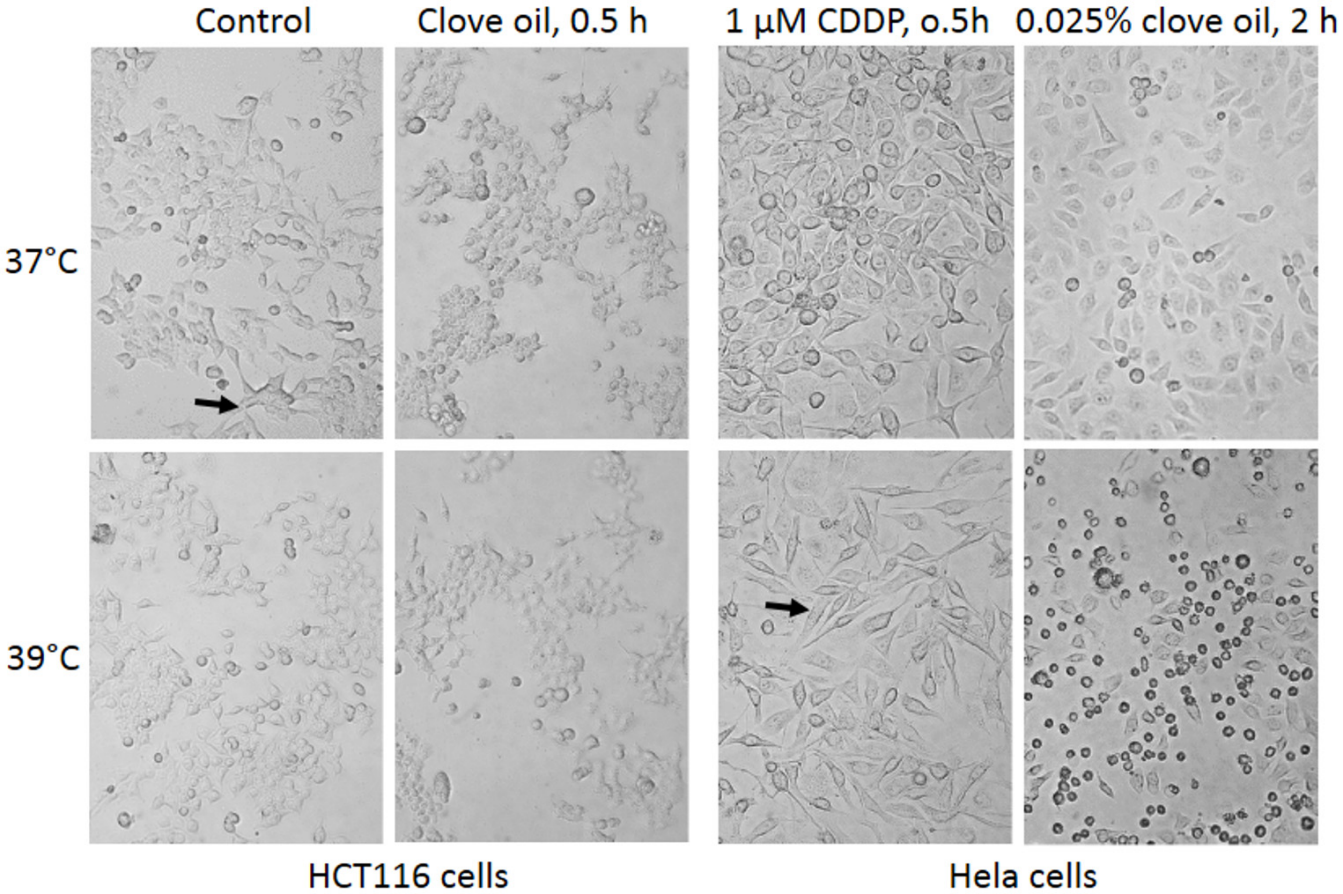
Effects of 39°C on cell morphology. **Left panel**: HCT116 cells at 37°C usually show irregular shape (arrow) but become spherical at 39°C. Treatment with 0.025% clove oil kills many more cells at 39°C than at 37°C, with the remaining cells shrunken to spherical shape within 30 minutes. **Right panel**: Thirty minutes post treatment with 1 μM cisplatin (CDDP), many Hela cells have changed to a spindle shape (arrow) at 39°C but remain unchanged at 37°C. Two hours post a treatment with 0.025% clove oil, many more Hela cells die at 39°C than at 37°C.

Hela, A549, H1650 and HEK293T cells did not display obvious difference in morphology between 37°C and 39°C. However, HCT116 cells were normally in an irregular shape at 37°C but many of them shrank to a spherical shape at 39°C (Fig 7). When treated with a low concentration (1 μM) of cisplatin, many Hela cells at 39°C, but not at 37°C, changed to spindle shape, which occurred as early as 30 minutes after the treatment (Fig 7). At a concentration of 0.025%, clove oil caused cell shrinkage and detachment in less than 30 minutes, which became more pronounced at 39°C two hours post the treatment (Fig 7). Likely, such quick changes may not involve cascades of gene activation and inactivation.

**Fig 8:**
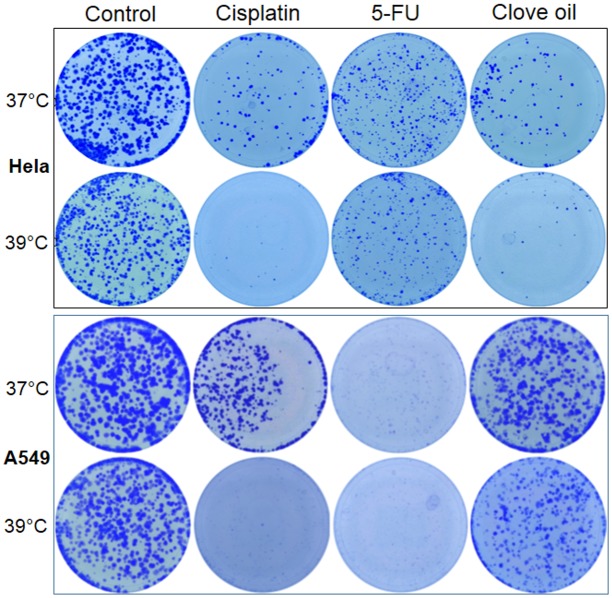
About 2000 Hela or A549 cells were seeded in each of 35-mm dishes and given 12 hours for the cells to attach the dish. The cells were then treated with 1 μM cisplatin, 4 μM 5-FU or 0.025% clove oil for 3 days. After the agent had been withdrawn, the cells were allowed to grow for one more week. Note that there are hardly any colonies in the 39°C dishes of the Hela and A549 cells treated with cisplatin, or of the A549 cells treated with clove oil, although one more week had been given for the growth, suggesting a post-treatment growth inhibition. However, this post-treatment effect at 39°C is not discerned in 5-FU treated Hela or clove oil treated A549 cells.

Interestingly, growth inhibition and cell death continued after cisplatin was withdrawn, manifested as further decrease in cell density or colony number determined by both microscopic observation and crystal violet staining, especially at 39°C and when the cells were seeded at a low density (Fig 8). These changes were more evidently manifested in HCT116 cells (Fig 3). Actually, there basically were no viable Hela, A549 and HECT116 cells left three days post cisplatin withdrawal in the 39°C dishes, while there still was a significant number of viable cells in the 37°C dishes. Obvious potentiation was also discerned when a 3-day treatment with cisplatin at 37°C was followed by 3 days of culture at 39°C (data not shown). These results suggest that the effects of 39°C on the chemotherapeutic effect of cisplatin is long-lasting, going beyond the treatment. However, this post-treatment effect of 39°C was both cell-line and agent specific, as it was not obvious in clove oil treated HCT116 cells (Fig 3) nor in 5-FU treated HCT116 and Hela cells (Figs 3 and 6).

### 39°C arrests cells at G1 phase and sometimes counteracts growth stimuli of serum

**Fig 9:**
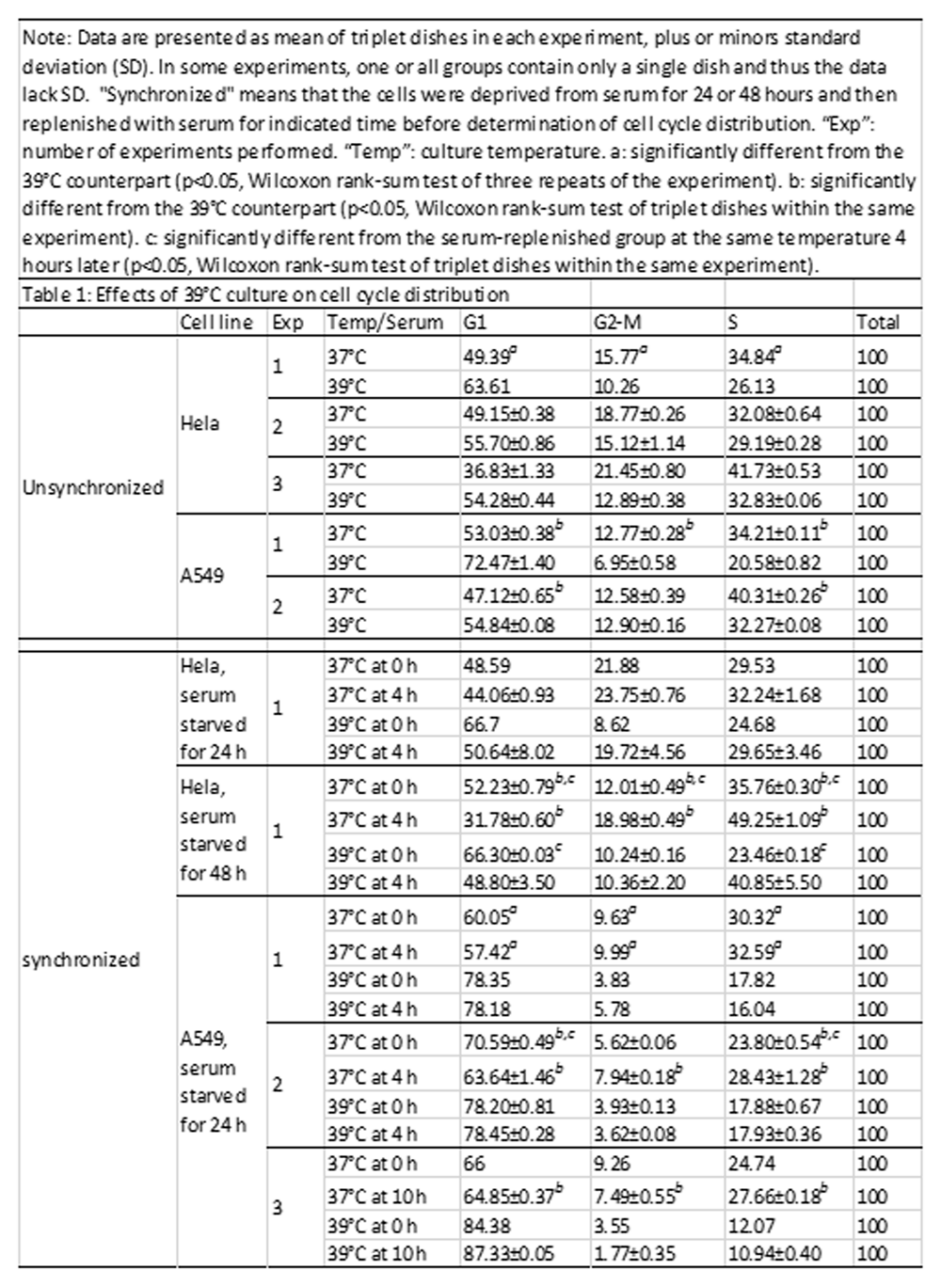
Effects of 39°C culture on cell cycle distribution. Data are presented as mean of triplet dishes in each experiment, plus or minors standard deviation (SD). In some experiments, one or all groups contain only a single dish and thus the data lack SD. "Synchronized" means that the cells were deprived from serum for 24 or 48 hours and then replenished with serum for indicated time before determination of cell cycle distribution. “Exp”: number of experiments performed. “Temp”: culture temperature. *a*: significantly different from the 39°C counterpart (p<0.05, Wilcoxon rank-sum test of three repeats of the experiment). *b*: significantly different from the 39°C counterpart (p<0.05, Wilcoxon rank-sum test of triplet dishes within the same experiment). *c*: significantly different from the serum-replenished group at the same temperature 4 hours later (p<0.05, Wilcoxon rank-sum test of triplet dishes within the same experiment).

**Fig 10:**
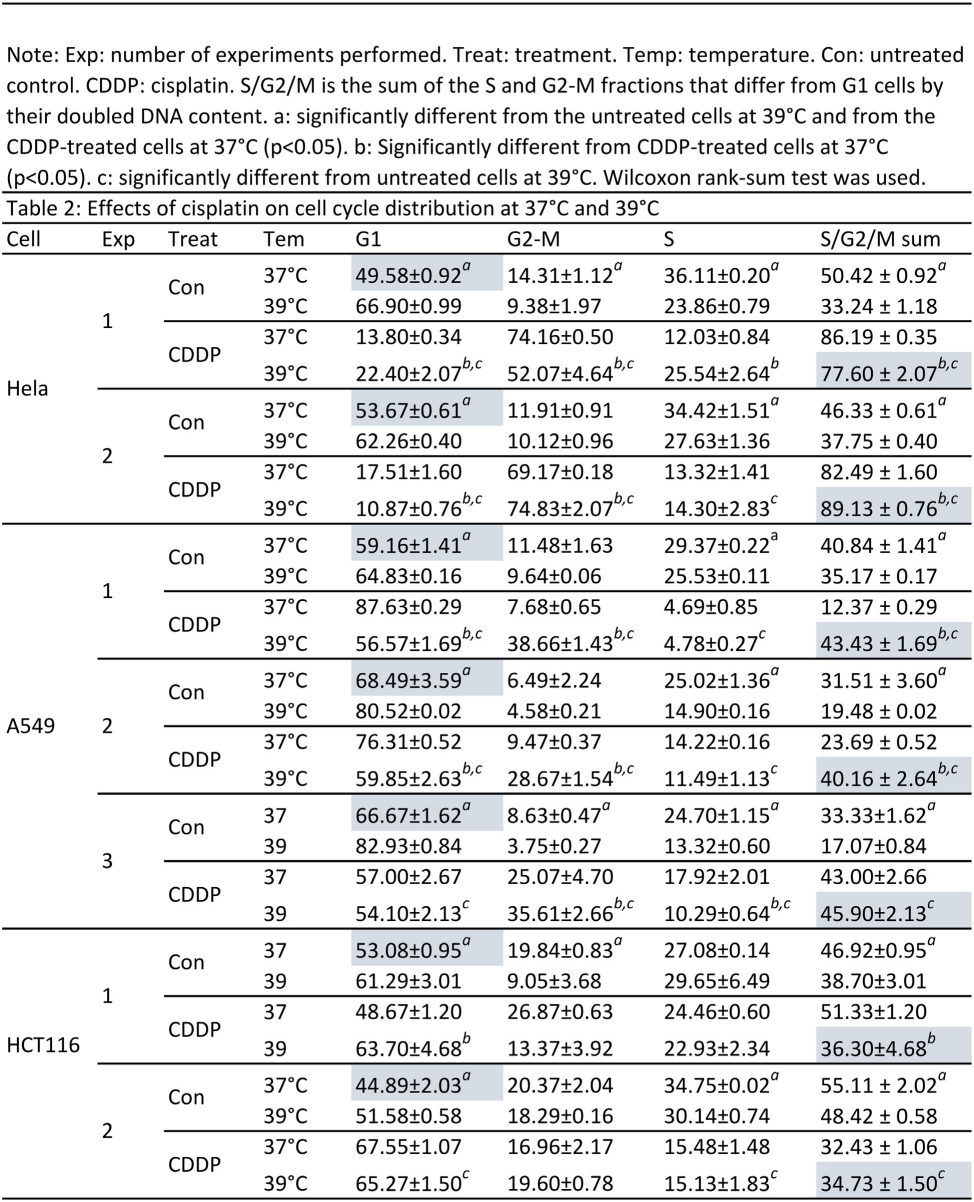
Effects of cisplatin on cell cycle distribution at 37°C and 39°C. Exp: number of experiments performed. Treat: treatment. Temp: temperature. Con: untreated control. CDDP: cisplatin. S/G2/M is the sum of the S and G2-M fractions that differ from G1 cells by their doubled DNA content. *a*: significantly different from the untreated cells at 39°C and from the CDDP-treated cells at 37°C (p<0.05). *b*: Significantly different from CDDP-treated cells at 37°C (p<0.05). *c*: significantly different from untreated cells at 39°C. Wilcoxon rank-sum test was used.

We allowed Hela and A549 cells to grow at 39°C for two weeks or longer with passages, so that the cells had adapted to the HT environment. The cells were then analyzed for cell cycle distribution. Because the data varied greatly among different experiments, the analyses were repeated for many times with most data shown in Fig 9. A larger G1 fraction and reciprocally smaller S and G2-M fractions at 39°C, compared with their 37°C counterparts, were constantly observed, thus confirming that the 39°C culture causes G1 arrest (Fig 9). Long-term cultured HCT116 cells were also analyzed, although as the controls for the cisplatin treatment, and the results also showed a G1 arrest at 39°C (Fig 10).

To determine whether cells at 39°C show a changed response to serum-derived growth stimuli, we withdrew Hela and A549 cells from serum for 24 hours, which widened the difference in the G1 fraction between 37°C and 39°C (Fig 9). As expected, four hours after serum replenishment, some G1-arrested Hela cells reentered the cell cycle, manifested as decreased G1 fraction and increased S and G2-M fractions, compared with the serum-deprived counterparts. Since it is well known, according to our experience and that of many others, that synchronization of Hela cells at G1 requires a longer serum starvation, we also deprived the cells from serum for 48 hours, which indeed further widened the difference in the G1 fraction between starved and replenished cells as expected (Fig 9). The difference between serum-starvation and -replenishment was also widened at 39°C, indicating that the effects of serum starvation and a higher temperature are additive on arresting the cells at G1.

Deprivation of A549 cells from serum also caused G1 arrest. Surprisingly, however, four or even ten hours after serum replenishment, the G1 fraction of A549 cells showed only a small decrease at 37°C, although this small decrease still widened the difference between serum starvation and replenishment and between 37°C and 39°C (Fig 9). More surprisingly, at 39°C, even 10 hours after serum replenishment, A549 cells were still not released from G1, indicating that the G1 arrest of A549 cells by 39°C cannot be overcome by growth stimuli of serum.

### 39°C and cisplatin alter cell cycle distribution in a cell-line specific manner

As expected, Hela, A549 and HCT116 cells treated with only the solvent showed G1 arrest at 39°C, manifested as increased G1 fraction and decreased S and G2-M fractions compared with their 37°C counterparts (highlighted in Fig 10). However, when treated with a low concentration (1 μM) of cisplatin, these cell lines exhibited different profiles of cell cycle distribution. Hela cells manifested a typical G2-M arrest, i.e. an increase in the G2-M fraction and a reciprocal decrease in the G1 fraction, at both 37°C and 39°C, compared with the untreated counterparts (Fig 10). Cisplatin-treated A549 cells constantly manifested a G2-M arrest at 39°C but often manifested G1 arrest at 37°C. Quite differently, cisplatin-treated HCT116 cells did not show any constant pattern of change in the cell cycle distribution either at 37°C or at 39°C, probably because in the absence of cisplatin at both temperatures, HCT116 cells already had a relatively high sum of the S/G2-M cells that differ from G1 cells by their doubled DNA content (Fig 10).

## Discussion

There have been plentiful studies published on HT therapy of cancer, but most of them determine only the effects of a short duration of HT, as short as minutes or hours [45–48]. Few studies have been performed on cell culture at a moderately feverish temperature for a period longer than one round of cell cycle, which is one day or longer for most cancer cell lines. For this reason, little information is available for evaluation of chronic HT effects on cell growth and cell cycle distribution. To our knowledge, the longest period of HT study in cell culture was performed by Heng’s lab, which shows that at a 42°C culture for two consecutive weeks, COLO-357 human pancreatic cancer cells manifest an initial growth inhibition but later resume growth, with increased cell death relative to the counterparts at the 37°C culture [28]. We study chronic influence of 39°C on cell growth and on the effects of several chemotherapeutic agents, which has clinical relevance based on two rationales: First, fever at this temperature occurs often in cancer and non-cancer patients and can last for days. Second, historically, doctors induced long duration of fever in patients to treat their cancers. For instance, some of the Coley’s patients were repeatedly induced to develop fever for years [18].

In conduction of this study, we realize that a higher temperature may increase the activities of MTT-reducing enzymes, which will result in a higher absorbance of reduced MTT and thus a spurious cell viability. Actually we once fell into this pitfall because it had not been well discussed in published studies of HT. Since some chemicals can alter the activity of the MTT-reducing enzymes [40], it is possible that some therapeutic chemicals may result in similar artifacts in MTT assays as well. Moreover, we also realize that we not only lack a feasible technique to quantify the effect of HT on cell growth in culture dishes but also lack good animal models to study in vivo effects of HT. Major constraints on animal study, besides the possible ethical concern on the animal protocol, include that in vivo effects largely involve the innate immunity and thus exclude the use of immunodeficient mice and that we lack good pyrogens to induce fever since commonly used pyrogens themselves, such as endotoxins [49,50], may have anticancer effects.

It has been known for a long time that usually cells need to be seeded at a threshold number or density in a culture dish, otherwise they will not grow. The explanation for this phenomenon is that cells need to collaborate with each other in order to survive and replicate, which is considered as the cell-culture version of the Allee effect [32,33]. One of our new findings is that some chemo drugs such as cisplatin and 5-FU affect this threshold, because when the cells are seeded in a low density, e.g. 1000–2000 cells per 35-mm dish, the difference in the colony number between chemo drug treated and untreated dishes is much greater, compared with the situation wherein over 20,000 cells were seeded in the dish. A higher temperature as one sort of treatment also affects this threshold by potentiating the effects of cisplatin and 5-FU on the cell-cell collaboration. Actually, there were basically no colonies of visible sizes left in the 39°C dish a few days post cisplatin withdrawal, whereas there remained a large number of colonies in the 37°C dish. Therefore, we have identified, for the first time, that cell-cell collaboration is a potential target of not only some chemotherapeutic agents, especially cisplatin, but also HT, and that the effects of these two factors on inhibition of cell-cell collaboration are additive. The mechanism behind this inhibition of cellular collaboration is unknown. Although the observed G1 arrest of cells seeded at a high density in the 39°C dishes may also occur and thus be a mechanism in the low-density dishes, we cannot confirm it because a low density culture does not provide enough cells for cell cycle analysis and for other mechanistic studies. Nevertheless, the G1 arrest of high-density cells at 39°C resembles the effect of serum deprivation but is milder as the growth inhibition cannot be discerned in the first a few days of culture. However, some cell lines such as A549, once arrested at G1 due to serum deprivation, are refractory to serum replenishment at 39°C, indicating that the growth inhibition by 39°C is potent enough to override the serum-derived growth stimuli to these cells. If these observations are directly translated to clinical oncology, we wonder whether after a surgical removal of a cancer mass, HT may be an effective approach to inhibit or even kill those cancer cells that have been peppered throughout some distant sites, resembling a low-density seeding, due to encroachments or metastases or simply because of the spread during the surgery. Such HT can happen clinically, because many patients experience post-operational fever, i.e. develop a fever after a surgery [51,52].

Usually, growth-arrested cells are resistant to chemotherapy, but, surprisingly, the HT-caused G1-arrest is associated with increased sensitivity to the therapeutic drugs we studied, probably because cells at a higher temperature have increased metabolisms, likely including increased biochemical activations of therapeutic chemicals. As introduced earlier in this paper, when animals including humans encounter a toxicant such as a chemotherapeutic agent, they usually decrease their metabolic rates, thus decreasing the body temperature, to minimize the toxicity [22]. Therefore, G1 arrest plus stimulation of metabolism by such as HT may be a good regimen to enhance chemotherapy. Supporting this conjecture, all three cancer cell lines studied, i.e. Hela, A549 and HCCT116, manifested not only G1 arrest but also increased sensitivity to cisplatin, 5-FU and clove bud extracts. Moreover, we observed that pre-culturing these cells at 39°C for several days before administration of these therapeutic agents seemed to be more effective (data not shown). However, when treated with cisplatin, these three cell lines showed different changes in the cell cycle distribution, both at 37°C and at 39°C. These changes may be results of cisplatin treatment and may not be the mechanisms for the additive or synergistic effects of cisplatin and HT. To many, but not all, cell lines, cisplatin is known to arrest cells at the G2-M and then kills the cells via mitotic catastrophe [45,53–55], which is also dubbed as mitotic-arrest-associated apoptosis but is actually a stress-induced cell death by our definition [8,56]. The cells are arrested at G2-M probably because they attempt to fix cisplatin-caused mutations. If so, those cancer cells that have significantly lost the mechanisms for DNA damage response and for DNA repair may not respond to cisplatin with increases in the S/G2-M fractions that differ from the G1 cells by their doubled DNA content.

Also interestingly, therapeutic effects of cisplatin on Hela, A549 and HCT116 cells are long -lasting, continuing beyond the cisplatin withdrawal, and this post-treatment effect is enhanced at 39°C as well. 5-FU and clove bud extracts also manifest similar post-treatment effects, but in an agent and cell-line specific manner. This observation raises a possibility that clinically the duration of certain chemotherapies may sometimes be shortened, i.e. be terminated before the tumor has regressed completely, especially when the patient experiences a fever fomented by the tumor *per se* or by treatment with a pyrogen such as interleukin-2 (IL-2). Indeed, IL-2, like IL-1, is a key mediator of the thermal-regulatory signaling activated by bacterial endotoxins and causing fever in patients with infectious diseases [57]. Although IL-2 has only been recommended for the treatments of melanoma and renal cancer [57,58], it has been combined with other agents to treat a spectrum of other cancers, including ovarian, breast, gastric, lung, colorectal, and head-and-neck cancers [59–63]. These treatments result in good outcomes, which may not only be due to a direct kill of the tumor cells by IL-2 but also be because the fever as its “side-effect” may enhance the efficacy of the other agents. Unfortunately, clinically IL-2 is often used together with antipyretics to prevent or alleviate fever.

Others and we have previously reported that ethanol extract of clove buds is a potent killer of a variety of cancer types in cell culture and in animals [34,43,44]. In the current study we further showed that a commercial essential oil of clove buds displayed potent therapeutic effects in the culture dish as well. The effects of the clove oil and ethanol extract on some cell lines are enhanced at 39°C and also persist beyond withdrawal of the extracts. At a relatively high concentration, the clove extracts kill cancer cells within 30 minutes, indicating that its acute cytotoxicity may not require a cascade of gene activation or inactivation.

In summary, we have observed in this study that 39°C culture causes a mild inhibition of cell replication via G1 arrest but in the meantime imposes onto the cells sensitivity to chemotherapies. The enhancement by 39°C is long-lasting, i.e. persisting beyond withdrawal of the chemotherapeutic agent, which to cisplatin seems to be universal for many cell lines but to 5-FU and clove bud extracts is agent and cell-line specific. Cellular mechanisms behind these effects may include an inhibition of cell-cell collaboration in the culture dish and an increase in cellular metabolisms at the higher temperature.
